# CD70 is a potential prognostic marker and significantly regulates cellular function in diffuse large B-cell lymphoma

**DOI:** 10.1371/journal.pone.0312445

**Published:** 2024-10-24

**Authors:** Kang Liu, Qiuyue Yang, Ping Liu, Kaibo Zhu, Min Zou, Qiang Zhu, Ping Yi, Kun Fang, Zimian Luo

**Affiliations:** 1 Hematology Laboratory, Central Hospital of Xiangtan, Xiangtan, China; 2 Department of Scientific Research Project, Wuhan Kindstar Medical Laboratory Co., Ltd., Wuhan, China; 3 Kindstar Global Precision Medicine Institute, Wuhan, China; 4 Department of Hematology, Central Hospital of Xiangtan, Xiangtan, China; European Institute of Oncology, ITALY

## Abstract

Extensive research has demonstrated that dysregulation of costimulatory molecule expression plays a pivotal role in cancer biology. However, the impact of intratumoral CD70 on the initiation, progression, and immune response in diffuse large B-cell lymphoma (DLBCL) remains poorly understood. This study aims to elucidate the clinical significance of CD70 in DLBCL diagnosis and prognosis, as well as its relationship with the immune microenvironment. We first analyzed CD70 expression across various cancers, including DLBCL, using multiple online databases (TIMER, GEPIA, GENT2, TNMPlot, GSCA, and GEO). We then evaluated the clinical correlations and prognostic value of CD70 in DLBCL. Additionally, we investigated the functional role of CD70 in DLBCL cells. Genomic alterations of CD70 were analyzed using the cBioPortal online tool. Co-expression network analysis was performed to assess the biological functions associated with CD70. Furthermore, we utilized TIMER2.0 to examine the correlation between CD70 expression and immune cell infiltration. Our results revealed that CD70 expression was significantly upregulated in DLBCL tissues compared to matched normal tissues, and high CD70 expression was associated with poor clinical outcomes in DLBCL patients. In vitro experiments demonstrated that CD70 inhibition promotes apoptosis and induces G1 phase arrest in DLBCL cells. Genomic alteration analysis showed that patients with CD70 alterations exhibited worse overall survival compared to those without such alterations. Co-expression and functional enrichment analyses indicated that CD70 is functionally related to tumor necrosis factor receptor binding and the NF-κB signaling pathway. Moreover, we found that CD70 expression levels were negatively correlated with B cell and NK cell infiltration in DLBCL. In conclusion, this study suggests that CD70 is a potential diagnostic and therapeutic biomarker for DLBCL. Our findings provide valuable insights for the development of novel therapeutic strategies targeting CD70 in DLBCL treatment.

## Introduction

Diffuse large B-cell lymphoma (DLBCL), the most prevalent subtype of Non-Hodgkin lymphoma, is a potentially curable hematological malignancy. The standard frontline treatment regimen consists of rituximab combined with cyclophosphamide, vincristine, doxorubicin, and prednisone (R-CHOP) [1]. However, only a subset of patients benefits from this regimen, with 30–40% remaining unresponsive or experiencing relapse after initial response [2]. Over the past decade, despite the more widespread use of autologous stem cell transplantation and significant efforts to improve upon R-CHOP as frontline therapy, including the addition of agents such as lenalidomide, bortezomib, or ibrutinib, the treatment of DLBCL remains an ongoing challenge. Consequently, novel therapeutic strategies are urgently needed to advance patient outcomes [3].

Exploring novel targets offers a promising avenue for advancing tumor therapy. CD70, a costimulatory molecule belonging to the tumor necrosis factor (TNF) superfamily, activates both innate and adaptive immunity through interaction with its ligand, CD27 [4]. Overexpression of CD70 has been observed in various hematologic and solid malignancies, playing a crucial role in cancer pathophysiology [5, 6]. Accumulating evidence has demonstrated the prognostic and therapeutic value of CD70 in malignant tumors, including acute myeloid leukemia [6, 7] and T-cell lymphomas [8, 9]. However, the function of intratumoral CD70 in the development, progression, and immune response of DLBCL remains largely unexplored.

In this study, we employed an integrated bioinformatics approach to analyze the expression, prognostic significance, and genomic alterations of CD70, as well as its biological function and correlation with immune cell infiltration in DLBCL. Our findings suggest that CD70 may contribute to the pathophysiological processes of DLBCL by modulating the tumor-immune microenvironment.

## Materials and methods

### Microarray data capture and processing

Gene expression microarray datasets (GSE56315, GSE10846, and GSE181063) were retrieved from the Gene Expression Omnibus (GEO) database (https://www.ncbi.nlm.nih.gov/geo/). The GSE56315 dataset, generated using the GPL570 platform, comprised 55 newly diagnosed DLBCL specimens and 33 matched normal specimens. The GSE10846 dataset, also from the GPL570 platform, included 181 DLBCL specimens treated with cyclophosphamide, doxorubicin, vincristine, and prednisone (CHOP) and 233 treated with R-CHOP. The GSE181063 dataset, collected using the GPL14951 platform, contained 1311 chemotherapy-treated DLBCL specimens. Raw microarray data were analyzed using R version 4.2.1 to assess gene expression levels and prognostic risk.

### Differential expression analysis of CD70 in diverse tumors

The differential gene expression profiles of CD70 in pan-cancer were obtained from the Tumor Immune Estimation Resource (TIMER, https://cistrome.shinyapps.io/timer/, a comprehensive database used for differential gene expression between tumor and adjacent normal tissues across all The Cancer Genome Atlas (TCGA) tumors), the Gene Expression Profiling Interactive Analysis (GEPIA, https://gepia.cancer-pku.cn/index/html, an interactive web server which can be used for evaluating RNA sequencing expression data for 9,736 tumor samples and 8,587 normal samples of TCGA and the Genotype-Tissue Expression (GTEx) database), the Gene Expression database of Normal and Tumor Tissues (GENT2; http://gent2.appex.kr/gent2) based on the expression profile from the U133_Plus_2.0 microarray platform (GPL570), the TNMplot (a database comprise 56,938 normal, tumor and metastatic samples from gene chip-based studies, TCGA, TARGET and GTEx databases), and the Gene Set Cancer Analysis (GSCA, https://bioinfo.life.hust.edu.cn/GSCA, an integrated database for genomic gene set cancer analysis). Additionally, CD70 expression profiles in variant cancers were downloaded from the UALCAN database (https://ualcan.path.uab.edu) and generated a bar graphic using GraphPad Prism software.

### CD70 expression and clinical correlation analysis in DLBCL

CD70 expression in DLBCL was evaluated using TCGA samples from GEPIA, GEO, and UALCAN databases, with the GSE56315 dataset used for verification. Inclusion criteria for DLBCL samples were: confirmed DLBCL diagnosis, available RNA-seq data, and complete baseline characteristics. Exclusion criteria were: normal tissue samples, metastatic samples, and incomplete baseline characteristics or expression profiles.

### Prognostic analysis of CD70 in DLBCL

The prognostic role of CD70 in DLBCL was assessed using least absolute shrinkage and selection operator (LASSO) regression, and univariate and multivariate Cox regression analyses. The GSE10846 and GSE181063 datasets were analyzed using R 4.2.1 to develop a CD70 prognostic risk model. Kaplan-Meier curves were used to validate the risk signature.

### Genetic alterations of CD70 in DLBCL and its downstream analysis

Genomic alterations of CD70 in DLBCL were analyzed using two datasets from PanCancer (n = 48) and DFCI, Nat Med 2018 (n = 135). The cBioPortal Oncoprint module visualized mutations, methylation, and mRNA expression alterations of CD70. Gene Ontology (GO) enrichment analysis was performed on differentially expressed genes. Kaplan-Meier survival analysis compared overall survival between DLBCL patients with and without CD70 genetic alterations. Clinical correlations of CD70 mutations and wild-type were examined.

### CD70 transcription factor prediction and co-expression gene analysis

Transcription factors associated with CD70 expression were predicted using the Cistrome DB database (http://cistrome.org/db/#/). GO functional enrichment and Kyoto Encyclopedia of Genes and Genomes (KEGG) pathway analyses were performed on predicted factors. A protein-protein interaction (PPI) network centered on CD70 was constructed using GeneMANIA (https://genemania.org/), followed by GO and KEGG pathway enrichment analyses.

### Correlation analysis of CD70 and its copy number alteration (CNA) with immune cell infiltration

TIMER2.0 was used to analyze the correlation between CD70 expression and immune cell infiltration using the CIBERSORT-ABS algorithm. TIMER was employed to evaluate the correlation between CD70 CNA and tumor-infiltrating immune cells in DLBCL.

### Prediction of CD70-targeting drugs

The Drug Gene Interaction Database (https://biogps.org/plugin/1136/dgidb) was used to predict potential drugs targeting CD70.

### Cell culture and transfection

The OCI-LY1 and OCI-LY3 cells were obtained from the cell bank of Hangzhou First Hospital in Zhejiang Province. The cells were cultured in complete IMDM (Hyclone, Cytiva), supplemented with 10% fetal bovine serum (FBS; Gibco, Thermo Fisher Scientific, Inc.), penicillin (100 U/mL), and streptomycin (100 μg/mL) (Hyclone, Cytiva), in a humidified incubator with 5% CO_2_ at 37°C. Three siRNAs targeting CD70 were designed and transfected into cells. Cells were seeded into a 24-well plate at a concentration of 2×10^5^ cells/well and cultured for 24 hours, followed by transfection with siRNA1, siRNA2, siRNA3, or a negative control (NC-siRNA) with a random sequence. The knockdown efficiency was assessed using qPCR, and the most effective siRNA was selected for subsequent experiments.

### Cell apoptosis detection

Transfected cells (1×10^6^ cells/time) were harvested and washed with ice-cold phosphate-buffered saline (PBS). The cell pellet was then resuspended in 1 mL of 1X Binding Buffer. Subsequently, 5 μL of Annexin V-FITC was added, and the mixture was incubated for 10 minutes at room temperature in the dark. Following this, 5 μL of propidium iodide (PI) was introduced, and the sample was further incubated for 5 minutes at room temperature, shielded from light. Flow cytometric analysis was performed within 1 hour of staining to assess cell viability and apoptosis.

### Cell cycle detection

The transfected cells were harvested by centrifugation at 1000 rpm for 5 minutes at 4°C. The cell pellet was then resuspended in 3 mL of pre-chilled PBS and centrifuged again under the same conditions. Subsequently, the cells were fixed by resuspension in pre-chilled 75% ethanol and incubated overnight at 4°C. Following fixation, the cells were washed with pre-chilled PBS and centrifuged as before. After discarding the supernatant, the cell pellet was resuspended in PI staining solution and incubated in the dark at 37°C for 30 minutes prior to flow cytometric analysis.

### Statistical analysis

Data obtained from various databases were statistically analyzed using GraphPad Prism version 7.0 (GraphPad Software Inc., LaJolla, CA, USA). The statistical significance of gene expression obtained from the TIMER database was evaluated using the Wilcoxon test. The ANOVA method was used for the comparison of gene expression obtained from the GEPIA database with the following threshold values: |log2FC| cutoff = 1, Log Scale = log2 (TPM + 1), and q-value cutoff = 0.01. A student’s *t*-test was performed for comparison between the two groups. Survival analysis was performed using the Kaplan-Meier method followed by log-rank tests. A *p*-value<0.05 was considered statistically significant.

## Results

### CD70 expression in pan-cancer

**Fig 1:**
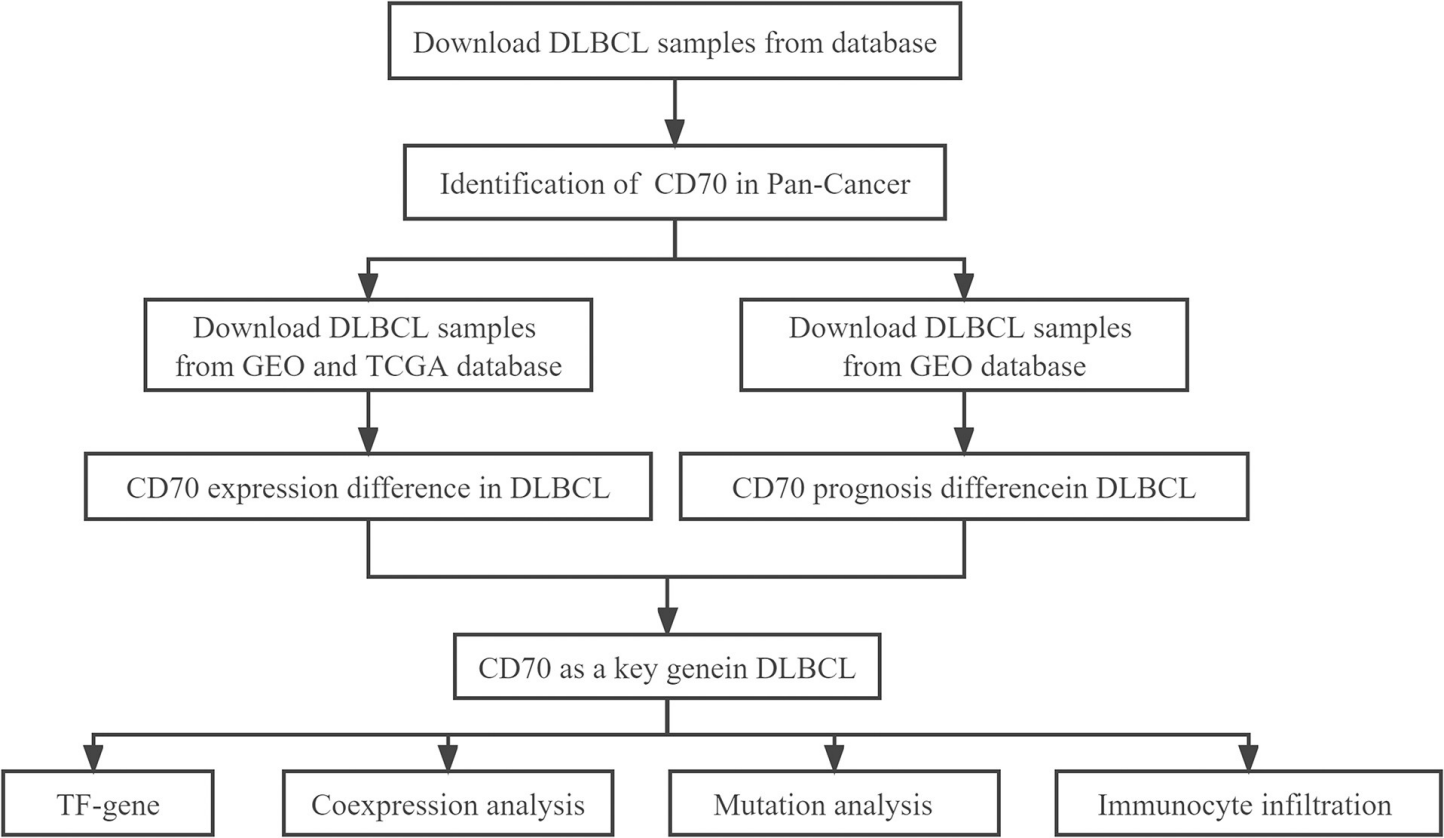
Flowchart of the study design.

**Fig 2:**
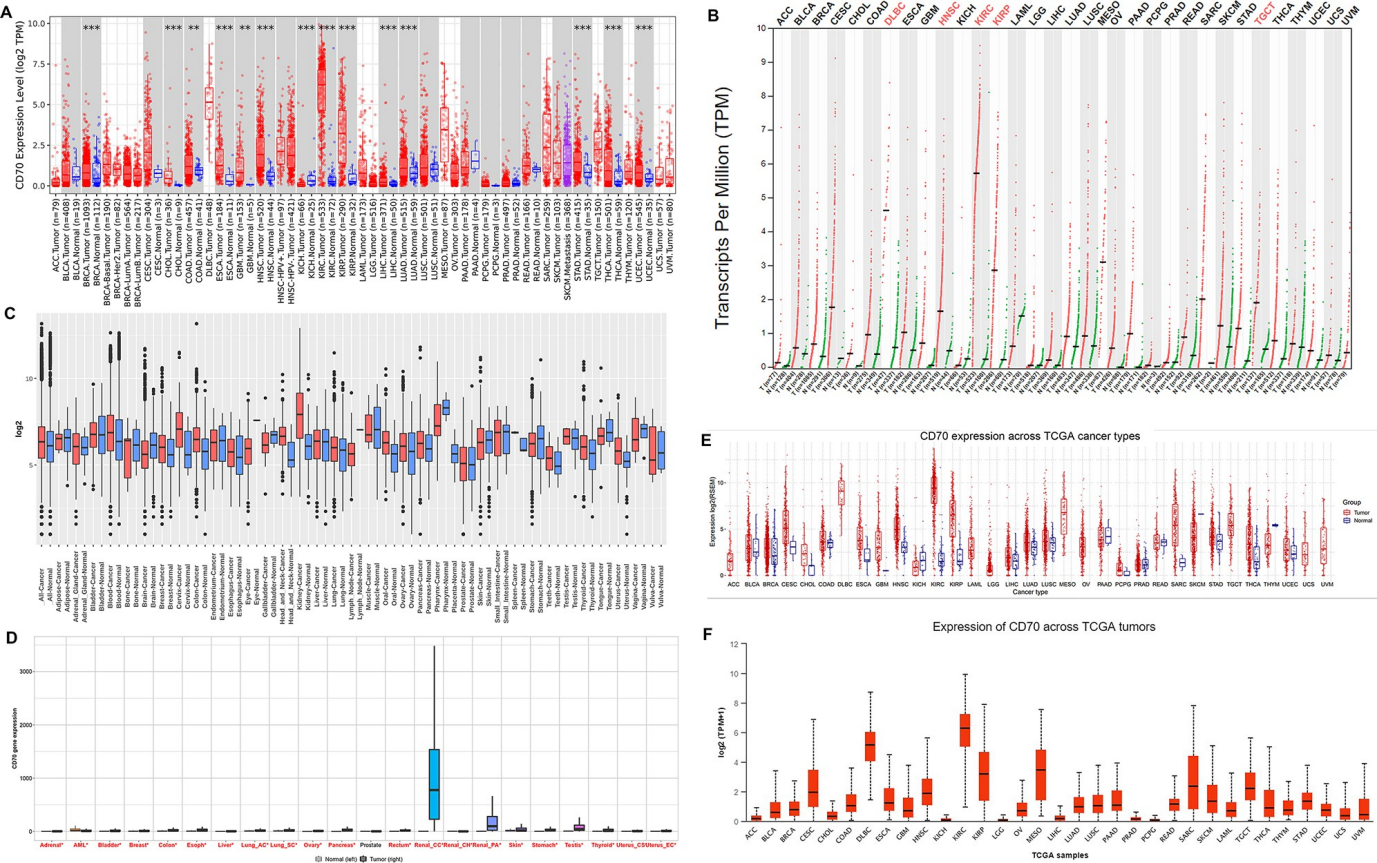
CD70 expression in pan-cancer analysis. CD70 expression levels in human cancers and adjacent normal tissues were evaluated using multiple databases: (A) TIMER, (B) GEPIA, (C) GENT2, (D) TNMplot, and (E) GSCA. CD70 expression was significantly different in most cancers compared to adjacent normal tissues. (F) Analysis of CD70 expression across various tumor types using the UALCAN database revealed that CD70 expression in diffuse large B-cell lymphoma (DLBCL) was significantly higher than in most other cancers. ** *p* < 0.01; *** *p* < 0.001. ACC: adrenocortical cancer; BLCA: bladder cancer; BRCA: breast invasive carcinoma; CESC: cervical squamous cell carcinoma and endocervical adenocarcinoma; CHOL: cholangiocarcinoma; COAD: colon adenocarcinoma; ESCA: esophageal carcinoma; GBM: glioblastoma multiforme; HNSC: head and neck squamous cell carcinoma; KICH: kidney chromophobe; KIRC: kidney renal clear cell carcinoma; KIRP: kidney renal papillary cell carcinoma; LAML: acute myeloid leukemia; LGG: brain lower grade glioma; LIHC: liver hepatocellular carcinoma; LUAD: lung adenocarcinoma; LUSC: lung squamous cell carcinoma; MESO: mesothelioma; OV: ovarian serous cystadenocarcinoma; PAAD: pancreatic adenocarcinoma; PCPG: pheochromocytoma and paraganglioma; PRAD: prostate adenocarcinoma; READ: rectum adenocarcinoma; SARC: sarcoma; SKCM: skin cutaneous melanoma; STAD: stomach adenocarcinoma; TGCT: testicular germ cell tumors; THCA: thyroid carcinoma; THYM: thymoma; UCEC: uterine corpus endometrial carcinoma; UCS: uterine carcinosarcoma; UVM: uveal melanoma.

This study evaluated the effect of intratumoral CD70 on the occurrence, progression, and immune response in DLBCL. The study design is illustrated in Fig 1. We analyzed CD70 expression across various tumor types and corresponding normal tissues using multiple online databases, including TIMER, GEPIA, GENT2, TNMPlot, and GSCA, all of which utilize data from TCGA datasets. RNA-sequencing data from the TIMER database revealed that CD70 was significantly upregulated in breast cancer, cervical squamous cell carcinoma and endocervical adenocarcinoma (CESC), breast invasive carcinoma (BRCA), cholangiocarcinoma (CHOL), colon adenocarcinoma (COAD), esophageal carcinoma (ESCA), glioblastoma multiforme (GBM), head and neck squamous cell carcinoma (HNSC), kidney renal clear cell carcinoma (KIRC), kidney renal papillary cell carcinoma (KIRP), liver hepatocellular carcinoma (LIHC), lung adenocarcinoma (LUAD), stomach adenocarcinoma (STAD), thyroid carcinoma (THCA), and uterine corpus endometrial carcinoma (UCEC). Conversely, CD70 expression was lower in KICH (Fig 2A). The GEPIA dataset demonstrated significantly increased CD70 expression in DLBC, CESC, HNSC, KIRC, and KIRP compared to normal tissues (Fig 2B). Analysis of the GENT2 database, using expression profiles from the U133_Plus_2.0 microarray platform (GPL570), showed significant upregulation of CD70 in breast, colon, head and neck, kidney, lung, oral, ovary, spleen, thyroid, and uterine cancers compared to adjacent normal tissues. In contrast, CD70 was notably downregulated in brain cancer (Fig 2C). Additional analyses using the TNMplot (Fig 2D) and GSCA (Fig 2E) databases further corroborated these findings, demonstrating significant differences in CD70 expression between human cancers and adjacent normal tissues. Furthermore, we compared CD70 expression across different tumor types using the UALCAN database, which revealed that CD70 expression in DLBCL was higher than in most other cancers (Fig 2F). Collectively, these results highlight the altered expression of CD70 in human cancers compared to normal tissues, with CD70 being overexpressed in most cancers, particularly in DLBCL.

### CD70 expression and clinical correlation analysis in DLBCL

**Fig 3:**
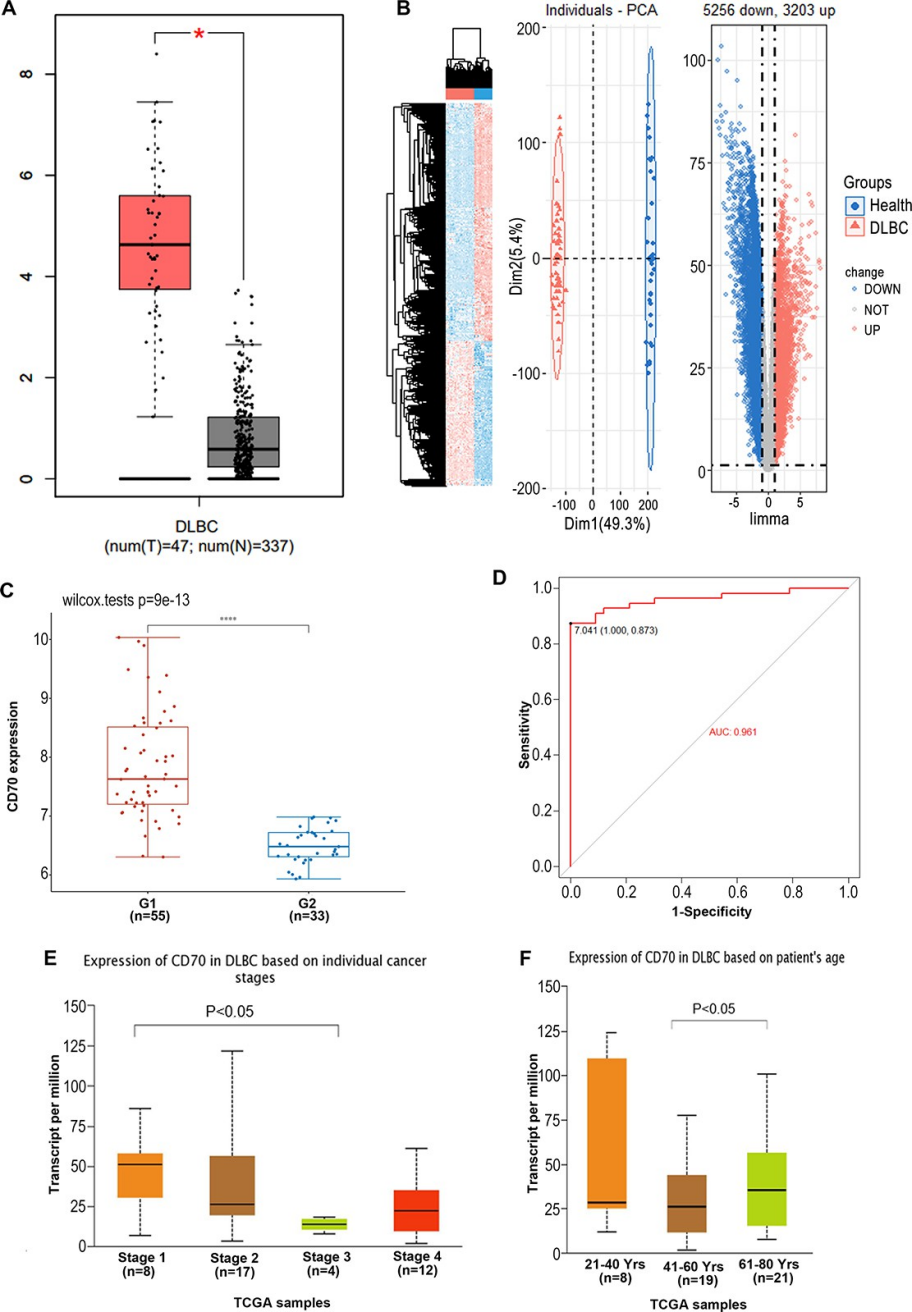
CD70 expression and clinical correlation analysis in DLBCL. (A) CD70 expression in DLBCL and normal tissues was evaluated using TCGA dataset. CD70 expression was significantly elevated in DLBCL compared to normal tissues. (B) Heatmap of differentially expressed genes in the GSE56315 dataset. Compared to healthy controls, 5,256 genes were downregulated and 3,203 genes were upregulated in DLBCL. (C) CD70 expression in DLBCL and normal tissues was assessed using the GSE56315 dataset, confirming significantly higher expression in DLBCL. (D) Receiver operating characteristic (ROC) curve analysis of CD70. The results suggest that CD70 is a potential diagnostic biomarker for DLBCL. (E) CD70 expression across different stages of DLBCL was evaluated using the UALCAN database. Notably, CD70 expression in stage I patients was significantly higher than in stage III patients. (F) CD70 expression in DLBCL patients of different age groups was analyzed using the UALCAN database. Middle-aged patients exhibited significantly lower CD70 expression compared to patients over 60 years old. * *p* < 0.05; **** *p* < 0.0001.

Based on the GEPIA database, we identified elevated CD70 expression in DLBCL compared to normal tissues. This finding was further corroborated by analysis of the TCGA dataset (Fig 3A), suggesting that CD70 may serve as a potential diagnostic marker for DLBCL. Additionally, we conducted differential expression analysis using the GSE56315 dataset, which confirmed the upregulation of CD70 in DLBCL (Fig 3B and 3C). Receiver operating characteristic (ROC) curve analysis demonstrated the diagnostic potential of CD70 for DLBCL, with an area under the curve (AUC) of 0.961 (Fig 3D). Furthermore, we examined the clinical correlations of CD70 expression in DLBCL using the TCGA dataset obtained from UALCAN. Our analysis revealed significant associations between CD70 expression and both clinical stage and patient age. Interestingly, CD70 expression was significantly higher in stage I patients compared to stage III patients (Fig 3E). Moreover, middle-aged patients exhibited markedly lower CD70 expression levels than patients over 60 years old (Fig 3F).

### Prognostic analysis of CD70 in DLBCL

**Fig 4:**
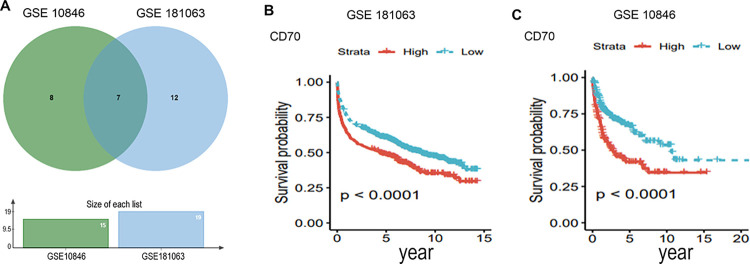
Prognostic analysis of CD70 in DLBCL. (A) Veen diagram of DLBCL-associated prognostic risk model data in GSE10846 and GSE181063 datasets. Seven risk signatures were obtained. The prognosis of CD70 in DLBC was analyzed using (B) GSE10846 and (C) GSE181063 datasets. The high expression of CD70 was associated with poor prognosis in DLBCL patients.

We conducted least absolute shrinkage and selection operator (LASSO) and univariate Cox regression analyses to develop a DLBCL-associated prognostic risk model using data from the GSE10846 and GSE181063 datasets. Initially, seven risk signatures were identified, including CD70. Subsequent multivariate Cox regression analysis excluded two of these signatures, leaving five in the final model (Fig 4A and Table 1). Kaplan-Meier analysis confirmed that high CD70 expression was significantly associated with poor prognosis in DLBCL patients treated in both the GSE10846 (Fig 4B) and GSE181063 (Fig 4C) cohorts.

**Table 1:** Univariable and multivariate Cox analysis results of GSE10846 and GSE181063 datasets.

		GSE10846		GSE181063	
Num	Gene	HR (univariable)	HR (multivariable)	HR (univariable)	HR (multivariable)
**1**	LTBR	0.26 (0.07–0.94, *p* = 0.04)	0.16 (0.04–0.71, *p* = 0.016)	0.45 (0.26–0.76, *p* = 0.003)	0.60 (0.33–1.09, *p* = 0.096)
**2**	TNFRSF1A	3.45 (1.07–11.11, *p* = 0.038)	4.60 (1.18–17.96, *p* = 0.028)	0.62 (0.40–0.98, *p* = 0.042)	0.81 (0.49–1.34, *p* = 0.407)
**3**	CD27	0.31 (0.12–0.84, *p* = 0.021)	0.39 (0.13–1.15, *p* = 0.089)	0.41 (0.25–0.66, *p* < 0.001)	0.46 (0.27–0.77, *p* = 0.003)
**4**	TNFRSF13B	5.88 (2.27–15.27, *p* < 0.001)	3.64 (1.34–9.87, *p* = 0.011)	2.05 (1.24–3.37, *p* = 0.005)	2.02 (1.24–3.28, *p* = 0.005)
**5**	CD70	3.26 (1.59–6.70, *p* = 0.001)	2.04 (1.00–4.20, *p* = 0.050)	2.26 (1.34–3.80, *p* = 0.002)	1.87 (1.11–3.16, *p* = 0.019)
**6**	EDAR	3.93 (1.16–13.35, *p* = 0.028)	1.63 (0.43–6.12, *p* = 0.468)	0.23 (0.09–0.57, *p* = 0.002)	0.31 (0.12–0.78, *p* = 0.013)
**7**	TMIGD2	0.43 (0.19–0.96, *p* = 0.040)	0.39 (0.16–0.94, *p* = 0.037)	0.27 (0.12–0.60, *p* = 0.001)	0.43 (0.19–0.98, *p* = 0.044)

HR, Hazard ratios.

### Genetic alterations of CD70 in DLBCL

**Fig 5:**
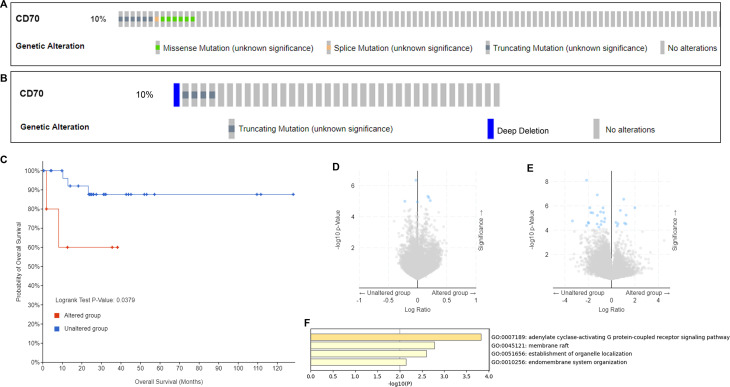
Genetic alterations of CD70 in DLBCL. Genomic alteration of CD70 in DLBCL was evaluated using datasets from (A) PanCancer and (B) DFCI, Nat Med 2018. CD70 was altered in 13 patients (10%) in the DFCI, Nat Med 2018 dataset, and 5 patients (10%) in the PanCancer dataset. (C) Prognostic analysis of CD70 in DLBCL was evaluated using PanCancer datasets. DLBCL patients with CD70 mutation showed poor prognosis compared to patients without CD70 mutation. (D) Methylation analysis of DLBCL was evaluated using PanCancer datasets. Compared with DLBCL patients with CD70 mutation, methylation levels of PKHD1, MAS1, and FIP1L1 were obviously increased, whereas RPL10A, MARCH10, FAM45A, and FAM45B were significantly decreased in DLBCL patients with CD70 mutation. (E) Differential gene analysis of DLBCL was evaluated using PanCancer datasets. 9 genes were remarkably upregulated, and 13 genes were notably downregulated in CD70 mutant DLBCL patients compared with CD70 wild-type patients. (F) GO functional enrichment of differential genes obtained in (E).

The cBioPortal online tool was employed to analyze genomic alterations of CD70 in two selected DLBCL datasets: PanCancer (n = 48) and DFCI, Nat Med 2018 (n = 135). As shown in Fig 5A, CD70 alterations were observed in 13 patients (10%) from the DFCI, Nat Med 2018 dataset, and in 5 patients (10%) from the PanCancer dataset (Fig 5B). We further conducted methylation, prognostic, and differential gene analyses using the PanCancer dataset due to its comprehensive clinical and genetic information. Prognostic analysis revealed that DLBCL patients harboring CD70 mutations exhibited significantly poorer prognosis compared to those without CD70 mutations (p = 0.0379, Fig 5C). Methylation analysis demonstrated that, relative to CD70 wild-type patients, CD70 mutant patients showed markedly increased methylation levels of PKHD1, MAS1, and FIP1L1, while methylation of RPL10A, MARCH10, FAM45A, and FAM45B was significantly decreased (Fig 5D). Differential gene expression analysis identified 9 genes that were significantly upregulated and 13 genes that were notably downregulated in CD70 mutant DLBCL patients compared to CD70 wild-type patients (Fig 5E). GO functional enrichment analysis of these differentially expressed genes revealed their primary involvement in the adenylate cyclase-activating G protein-coupled receptor signaling pathway (Fig 5F). Additionally, clinical correlation analysis uncovered significant differences between CD70 mutant and wild-type patients in terms of ICD-10 classification, International Classification of Diseases for Oncology, Third Edition (ICD-O-3) site code, and MSIsensor score (Table 2).

**Table 2:** Clinical correlation analysis between DLBCL patients with and without CD70 mutation.

Clinical Attribute	Attribute Type	Statistical Test	*p*-Value
**ICD-10 Classification**	Patient	Chi-squared Test	3.52E-04
**International Classification of Diseases for Oncology, Third Edition ICD-O-3 Site Code**	Patient	Chi-squared Test	0.0233
**MSIsensor Score**	Sample	Wilcoxon Test	0.0405
**TMB (nonsynonymous)**	Sample	Wilcoxon Test	0.056
**Mutation Count**	Sample	Wilcoxon Test	0.056
**MSI MANTIS Score**	Sample	Wilcoxon Test	0.182
**Race Category**	Patient	Chi-squared Test	0.246
**Last Communication Contact from Initial Pathologic Diagnosis Date**	Patient	Wilcoxon Test	0.322
**Other Patient ID**	Patient	Chi-squared Test	0.423
**Fraction Genome Altered**	Sample	Wilcoxon Test	0.477
**Tumor Type**	Sample	Chi-squared Test	0.631
**Radiation Therapy**	Patient	Chi-squared Test	0.647
**Aneuploidy Score**	Sample	Wilcoxon Test	0.688
**New Neoplasm Event Post Initial Therapy Indicator**	Patient	Chi-squared Test	0.703
**Person Neoplasm Cancer Status**	Patient	Chi-squared Test	0.727
**Sex**	Patient	Chi-squared Test	0.743
**Tissue Prospective Collection Indicator**	Sample	Chi-squared Test	0.796
**Tissue Retrospective Collection Indicator**	Sample	Chi-squared Test	0.796
**Tissue Source Site Code**	Sample	Chi-squared Test	0.806
**Tissue Source Site**	Sample	Chi-squared Test	0.806
**Form completion date**	Patient	Chi-squared Test	0.837
**Patient Weight**	Patient	Wilcoxon Test	0.859
**Diagnosis Age**	Patient	Wilcoxon Test	0.876
**Birth from Initial Pathologic Diagnosis Date**	Patient	Wilcoxon Test	0.894
**Ethnicity Category**	Patient	Chi-squared Test	0.901
**American Joint Committee on Cancer Publication Version Type**	Patient	Chi-squared Test	0.939

### CD70 transcription factor prediction and co-expression network analysis

**Fig 6:**
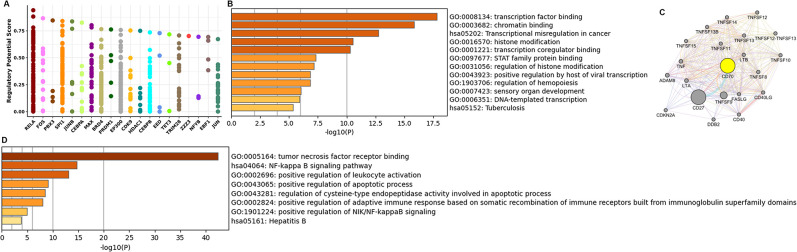
CD70 transcription factor prediction and co-expression network analysis. (A) The top 20 transcription factors that influenced the transcription of CD70 in Cistrome DB database. (B) GO and KEGG enrichment analysis of the transcription factors. (C) A protein-protein interaction network centered on CD70 was analyzed using GeneMANIA. (D) GO and KEGG enrichment analysis based on the obtained genes.

To elucidate the biological function of CD70, we utilized the Cistrome DB database to predict transcription factors influencing CD70 transcription. Fig 6A displays the top 20 transcription factors with the highest scores, including RELA, FOS, PBX3, and SPI1. GO enrichment analysis revealed that these transcription factors are primarily associated with transcription factor binding, chromatin binding, histone modification, transcription coregulatory binding, and STAT family protein binding. KEGG pathway enrichment analysis indicated that these factors are predominantly involved in transcriptional misregulation in cancer pathways (Fig 6B). Furthermore, we constructed a PPI network centered on CD70 using GeneMANIA (Fig 6C). GO and KEGG enrichment analyses of the genes within this network revealed significant enrichment in GO terms such as tumor necrosis factor receptor binding and positive regulation of leukocyte activation. The most significantly enriched KEGG pathway was the NF-κB signaling pathway (Fig 6D).

### Correlation analysis between CD70 CNA and immune cell infiltration

**Fig 7:**
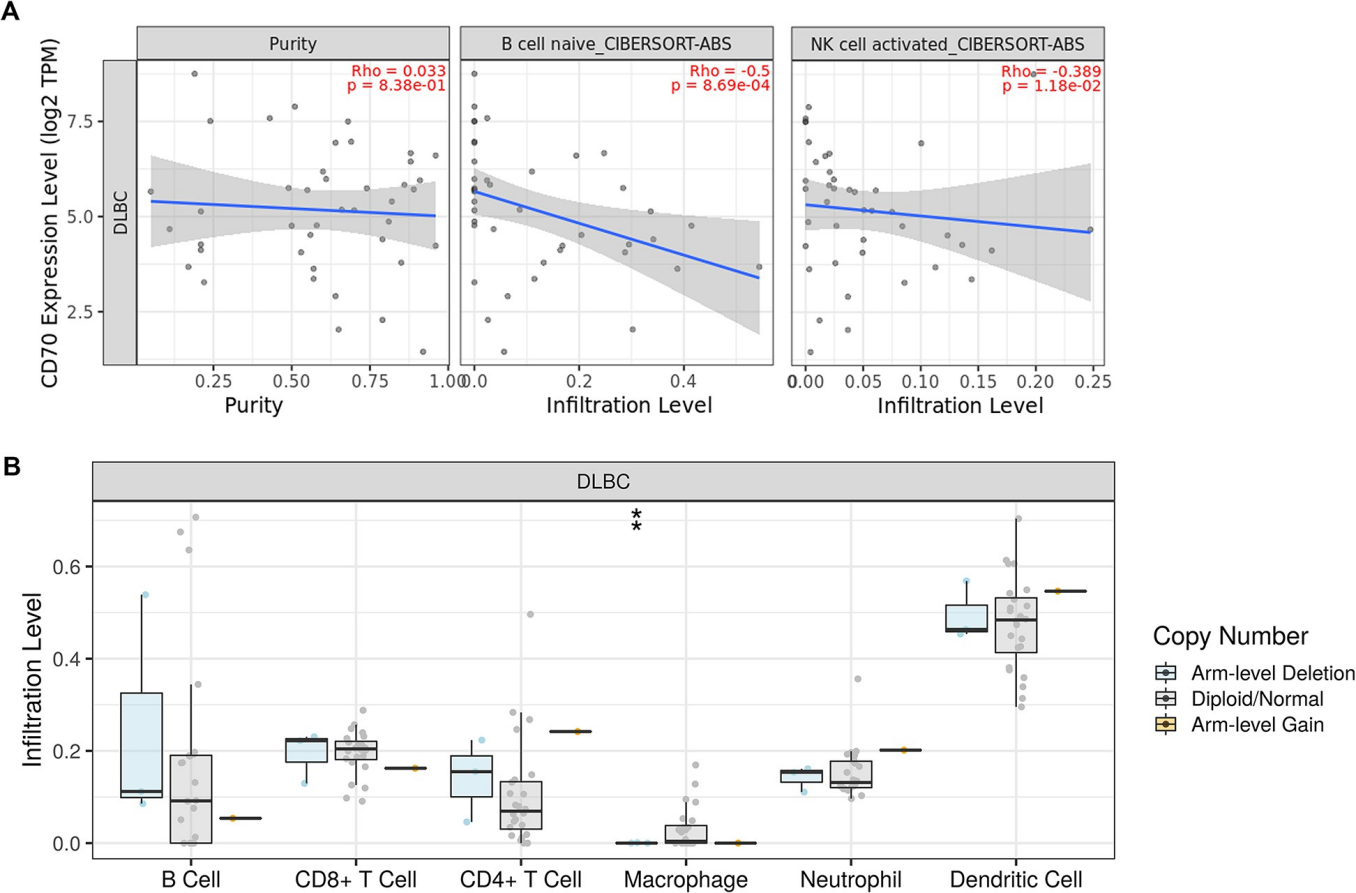
Correlation analysis between CD70 copy number alteration and immune cell infiltration. (A) TIMER2.0 was used to analyze the correlation between CD70 and immune infiltrated cells using the CIBERSORT-ABS algorithm. The expression level of CD70 was negatively correlated with B and NK cell infiltration in DLBCL. (B) TIMER was used to evaluate the correlation between CD70 copy number alteration and several tumor-infiltrating immune cells in DLBCL. The deletion of the CD70 copy number was significantly associated with the low macrophage infiltration. ** *p* < 0.01.

The tumor microenvironment has been established as a critical factor in tumorigenesis. Our current study revealed a significant negative correlation between CD70 expression levels and the infiltration of B and NK cells in DLBCL (p < 0.05, Fig 7A). Moreover, we observed that CD70 copy number deletion was strongly associated with reduced macrophage infiltration (Fig 7B).

### Targeted drug prediction of CD70

Moreover, we identified potential CD70-targeting drugs using the DGIdb. Our analysis revealed that MDX1411 and SGN-75 (vorsetuzumab mafodotin) are promising candidates for targeting CD70. Currently, several drugs have been developed to treat patients with CD70-positive malignancies, including SGN-CD70A [10], ARGX-110 [11], SGN-75 [12], and BR108.

### CD70 inhibition promotes apoptosis in OCI-LY1 and OCI-LY3 cells

**Fig 8:**
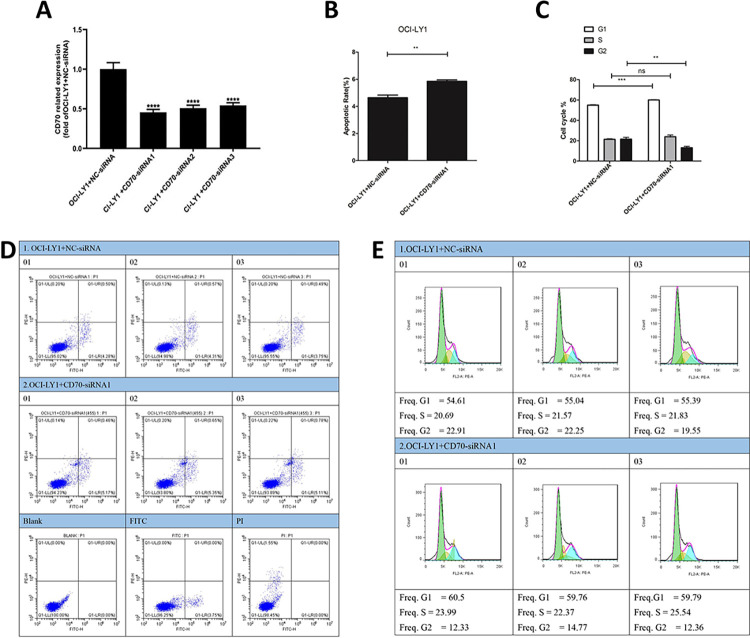
Functional analysis of CD70 in the DLBCL cell line OCI-LY1. (A) OCI-LY1 cells were transfected with CD70-siRNA1, CD70-siRNA2, CD70-siRNA3, or the negative control (NC-siRNA), and qPCR was performed to detect the interference effect. (B, D) Flow cytometry was performed to evaluate the apoptosis rate of OCI-LY1 cells transfected with CD70-siRNA1 or NC-siRNA. (C, E) Flow cytometry was performed to evaluate the cell cycle of OCI-LY1 cells transfected with CD70-siRNA1 or NC-siRNA. ** *p* < 0.01; *** *p* < 0.001; **** *p* < 0.0001; ^ns^ *p* > 0.05.

**Fig 9:**
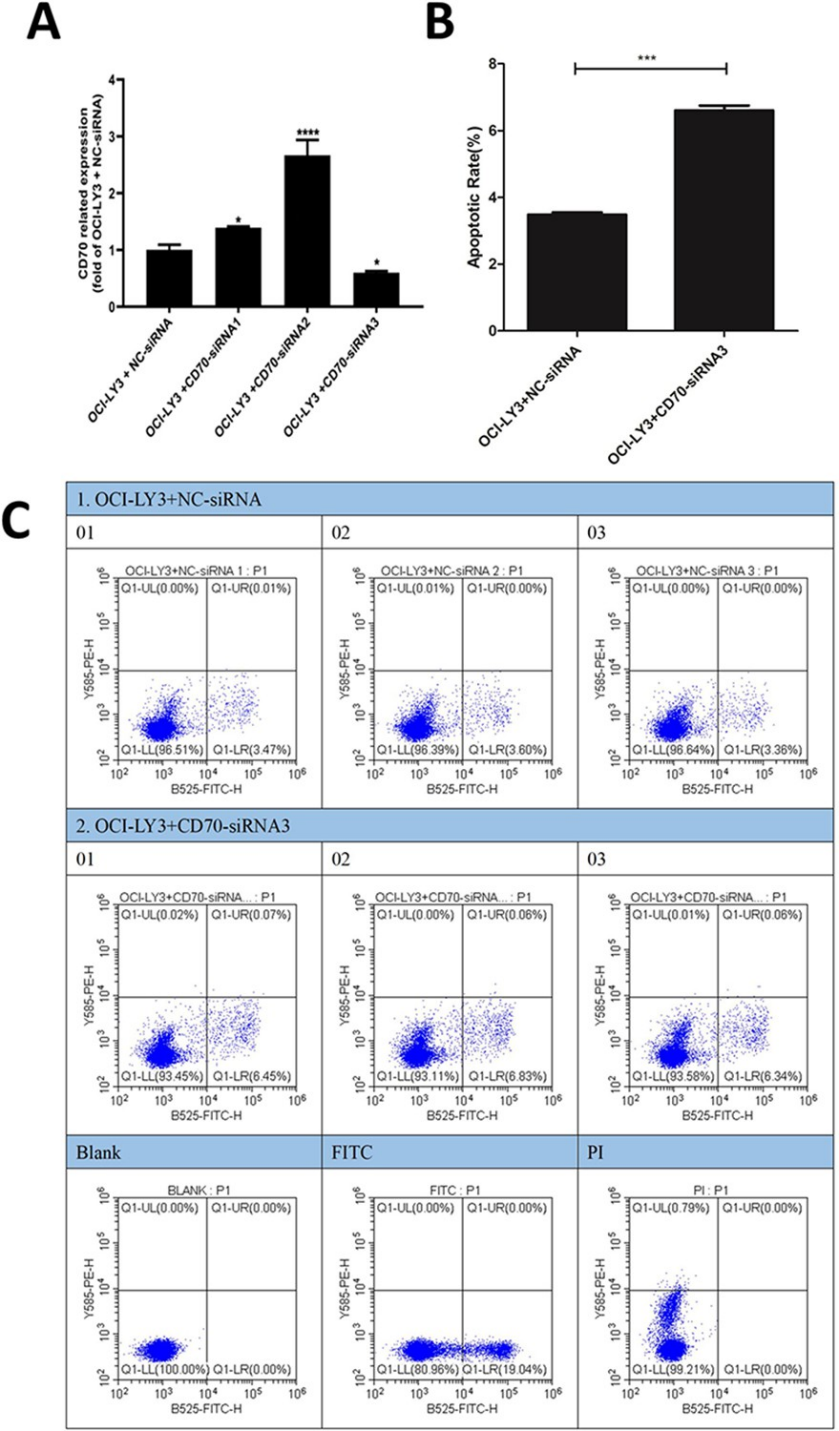
Functional analysis of CD70 in the DLBCL cell line OCI-LY3. (A) OCI-LY3 cells were transfected with CD70-siRNA1, CD70-siRNA2, CD70-siRNA3, or the negative control (NC-siRNA), and qPCR was performed to detect the interference effect. (B, C) Flow cytometry was performed to evaluate the apoptosis rate of OCI-LY3 cells transfected with CD70-siRNA3 or NC-siRNA. * *p* < 0.05; *** *p* < 0.001; **** *p* < 0.0001.

To investigate the function of CD70 in DLBCL, we inhibited its expression in the GCB-DLBCL cell line OCI-LY1 and the ABC-DLBCL cell line OCI-LY3. The qPCR results demonstrated that CD70-siRNA1, CD70-siRNA2, and CD70-siRNA3 significantly suppressed CD70 expression compared to the negative control siRNA (NC-siRNA), with CD70-siRNA1 exhibiting the most potent interference effect (Fig 8A). Consequently, siRNA1 was selected for subsequent experiments. Apoptosis assays revealed a significant increase in the apoptosis rate in the CD70-siRNA1 group compared to the NC-siRNA group (Fig 8B and 8D). Furthermore, cell cycle analysis indicated that CD70 inhibition led to G1 phase arrest and a reduction in the G2 phase population of OCI-LY1 cells (Fig 8C and 8E). To further corroborate the role of CD70 in DLBCL, similar findings were observed in the ABC-subtype cell line OCI-LY3. Compared to the NC-siRNA group, the CD70 expression level was significantly lower in the CD70-siRNA3 group (Fig 9A). Therefore, siRNA3 was selected for subsequent experiments. Apoptosis detection results demonstrated that the apoptosis rate increased significantly in the CD70-siRNA3 group compared to the NC-siRNA group (Fig 9B and 9C). These findings strongly suggest that CD70 inhibition promotes apoptosis and induces cell cycle arrest in DLBCL cells.

## Discussion

Carcinogenesis is a complex, multistep process influenced by various physiological conditions, including differential gene expression, genetic alterations, mutations, and the immune microenvironment [13, 14]. Thus, elucidating the molecular mechanisms of tumorigenesis from multiple perspectives is crucial for developing novel therapeutic strategies. In this study, we analyzed CD70 expression in various cancers, including DLBCL, using TIMER, GEPIA, GENT2, TNMPlot, GSCA, and GEO online databases. Our findings demonstrated significantly elevated CD70 expression in DLBCL tissues compared to matched normal tissues. Furthermore, ROC analysis identified CD70 as a potential diagnostic marker for DLBCL. Bertrand et al. reported that CD70 was markedly upregulated in both germinal center B-cell-like and activated B-cell-like DLBCL compared to normal tissue, with elevated CD70 expression associated with shorter overall survival in both subtypes [15]. Interestingly, our analysis revealed significantly higher CD70 expression in stage I patients compared to stage III patients. However, Kaplan-Meier analysis of both the GSE10846 and GSE181063 datasets showed that high CD70 expression was associated with poor prognosis in DLBCL patients treated with chemotherapy. This apparent discrepancy suggests that high CD70 expression might be linked to chemotherapy resistance or chemosensitivity, warranting further investigation.

Genetic alterations and mutations are considered potent drivers of cancer development, including DLBCL [16, 17]. We analyzed genetic alterations and mutations of CD70 in two DLBCL datasets from PanCancer (n = 48) and DFCI, Nat Med 2018 (n = 135), finding that 10% of DLBCL patients exhibited CD70 alterations in both datasets. Patients with CD70 alterations demonstrated worse overall survival compared to those without such alterations. Additionally, we observed changes in methylation levels of several genes in DLBCL patients with CD70 alterations. These results underscore the importance of considering CD70 genetic alterations or mutations when evaluating its prognostic value in DLBCL.

PPI network and functional enrichment analyses of CD70 were performed. GO enrichment analysis revealed that CD70 was associated with the regulation of leukocyte activation, apoptotic processes, adaptive immune responses, and NIK/NF-kappaB signaling. KEGG pathway enrichment analysis showed that the CD70 PPI network was primarily associated with the NF-kappa B signaling pathway. The involvement of NF-kappa B signaling in tumor development has been extensively documented [18]. Previous studies have shown that, without stimulation, NF-kappa B dimers remain in an inactive state through binding to I-kappa B. Upon stimulation, NF-kappa B modulates gene expression affecting various biological processes, including inflammation, cell survival, proliferation, and immune responses in numerous cancers [19, 20]. These findings suggest that CD70 might be involved in DLBCL pathophysiology through regulation of cell survival and the tumor-immune microenvironment. Furthermore, our functional studies in the GCB-DLBCL cell line OCI-LY1 and the ABC-DLBCL cell line OCI-LY3 demonstrated that CD70 inhibition promotes apoptosis.

As integral components of the tumor microenvironment, immune infiltrates play a crucial role in cancer progression, responses to immunotherapy, and clinical outcomes [21]. Numerous studies have investigated correlations between tumor clinical outcomes and levels of immune cell infiltration, identifying several cell types as prognostic markers, including NK cells, T cells, and B cells [22]. Our current study found that CD70 expression levels were negatively correlated with B and NK cell infiltration in DLBCL. Plonquet et al. reported that NK cell numbers correlated with clinical outcomes in DLBCL, with low NK cell counts associated with shorter event-free survival [23]. The role of intratumoral B cells in cancer remains unclear. Evidence has shown that NK cell infiltration is associated with a good prognosis in some histological subtypes of epithelial ovarian and breast cancers [24, 25]. Collectively, these results suggest that CD70 might play a fundamental role in DLBCL clinical outcomes by regulating the tumor-immune microenvironment.

## Conclusion

In conclusion, our comprehensive analysis of CD70 expression and function in DLBCL utilizing multiple online databases revealed significant findings. CD70 was notably upregulated in DLBCL tissues, and its high expression correlated with poor clinical outcomes in DLBCL patients. Inhibition of CD70 promoted apoptosis and induced G1 phase arrest in DLBCL cells. Both co-expression network and immune cell infiltration analyses suggested that CD70 may contribute to DLBCL pathophysiology by modulating the tumor-immune microenvironment. While our study primarily relied on in silico analyses of existing datasets, these findings provide a strong foundation for further experimental validation. Additional in vitro and in vivo studies are warranted to substantiate and expand upon our conclusions. Nonetheless, our results identify CD70 as a promising diagnostic and therapeutic biomarker for DLBCL, offering valuable insights for developing targeted treatment strategies.
